# Single cell imaging of Bruton's Tyrosine Kinase using an irreversible inhibitor

**DOI:** 10.1038/srep04782

**Published:** 2014-04-24

**Authors:** Anna Turetsky, Eunha Kim, Rainer H. Kohler, Miles A. Miller, Ralph Weissleder

**Affiliations:** 1 Center for Systems Biology, Massachusetts General Hospital, 185 Cambridge St, CPZN 5206, Boston, MA 02114; 2 Department of Systems Biology, Harvard Medical School, 200 Longwood Ave, Boston, MA 02115; 3 These authors contributed equally to this work.

## Abstract

A number of Bruton's tyrosine kinase (BTK) inhibitors are currently in development, yet it has been difficult to visualize BTK expression and pharmacological inhibition in vivo in real time. We synthesized a fluorescent, irreversible BTK binder based on the drug Ibrutinib and characterized its behavior in cells and in vivo. We show a 200 nM affinity of the imaging agent, high selectivity, and irreversible binding to its target following initial washout, resulting in surprisingly high target-to-background ratios. In vivo, the imaging agent rapidly distributed to BTK expressing tumor cells, but also to BTK-positive tumor-associated host cells.

Bruton's tyrosine kinase (BTK) is a non-receptor tyrosine kinase with restricted cellular expression largely limited to B lymphocytes, macrophages/monocytes, and certain cancer cells^1^,^2^,^3^,^4^. As a critical component of the B cell receptor (BCR) signaling network, BTK is crucial for B cell development^5^,^6^ and acts in multiple anti-apoptotic signaling pathways, including the PI3K-AKT^7^, STAT5^8^ and NF-κB^9^,^10^ pathways. BTK is thus intimately involved in regulating cell survival, proliferation, and differentiation. In human haematological malignancies, BTK is abundantly expressed and activated in malignant cells from patients with B-cell multiple myeloma^11^, acute myeloid leukemia (AML)^12^, chronic lymphocytic leukemia (CLL)^13^, and non-Hodgkin's lymphoma (NHL)^14^,^15^. It is thus estimated that there are about 80,000 new BTK-positive haematologic malignancies in the US per year.

Several BTK inhibitors are under development and have shown remarkable efficacy in early clinical trials^16^,^17^,^18^,^19^,^20^. Ibrutinib (PCI-32765) is one example of a selective, irreversible BTK inhibitor, whose covalent binding results in long-lasting target occupancy, retaining inhibitory effect until new protein is synthesized^21^,^22^. The irreversible inhibitory effect of Ibrutinib is attributed to an electrophilic group on the molecule binding covalently to Cys 481 in the active site of BTK^23^. Most clinical trials to date have relied on insensitive standardized Response Evaluation Criteria approaches, such as computed tomography (CT), to image drug effects, while a denaturing gel electrophoresis assay has been used when tissue is available in Ibrutinib trials^21^,^24^. In the latter assay, a fluorescent probe binds any unoccupied BTK in tissue biopsy or blood to produce a fluorescent band; the lighter the band, the more BTK is occupied by drug. Even in co-clinical trials using mouse models, drug efficacy is largely tested by volumetrics or cell counts, while little is known about the kinetics of drug distribution *in vivo*, accumulation across cell types, and their respective heterogeneities or drug effects. There is therefore a need for imaging tools to study BTK inhibitor distribution at the single cell level *in vivo*. Such tools could be valuable to better understand kinetics, selectivity, drug action, inform on dose ranging studies, and allow in vitro testing of harvested cells from patients. Furthermore, imaging would be especially useful in the development of next generation BTK inhibitors^19^,^25^,^26^.

We hypothesized that an Ibrutinib-like scaffold could be converted into a companion diagnostic imaging agent by modification with a fluorescent tag while preserving irreversible target binding. The goal of the current study was to explore whether terminal modification of Ibrutinib could generate a BTK-selective imaging agent for *in vivo* use. Given the irreversible nature of target binding, one would expect improved target-to-background ratios following the clearance of unbound fractions. We indeed show remarkable target localization, specificity, and the ability to measure drug distribution and target inhibition *in vivo*. As more attention is paid to cell-to-cell heterogeneity in drug response and its impact on efficacy, we believe this will be a useful tool to study BTK expression and inhibition^27^.

## Results

**Figure 1:**
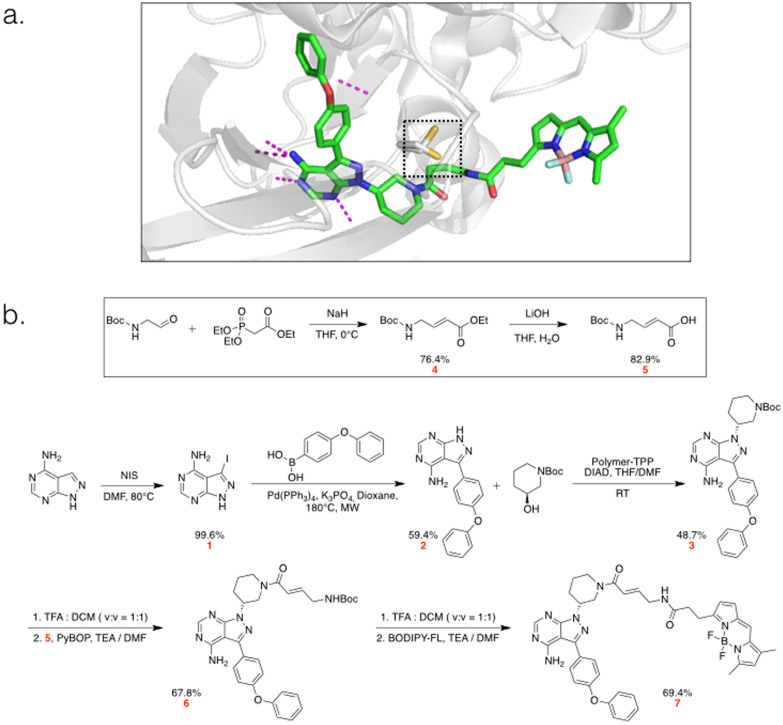
Structure and Synthetic Scheme of Ibrutinib-BFL (7). a. Crystal structure prediction of Ibrutinib-BFL (**7**) in its binding pocket of BTK. The reactive cysteine is highlighted in yellow inside the box. Hydrogen bonds are shown as purple dotted lines. 3D models were rendered using PyMol. b. Synthetic scheme of Ibrutinib-BFL (**7**).

We sought to design a bright, fluorescent derivative of an irreversible BTK inhibitor that would preserve the selectivity of the parent drug. As such a model agent, Ibrutinib fits tightly into the ATP binding pocket of BTK, forms an irreversible bond with Cys481, and has a suitable modification point for fluorochrome attachment (PDB: 3GEN, Fig. 1a). A BODIPY-FL modified Ibrutinib (Ibrutinib-BFL) was designed and synthesized de novo in seven steps (Fig. 1b). Briefly, iodination of commercially available pyrazolopyrimidine compound with N-Iodosuccinimide, followed by Suzuki coupling of the product with 4-phenyloxybenzene boronic acid, resulted in compound **2**. Mitsunobu reaction of compound **2** with N-Boc-3-hydroxypiperidine resulted in compound **3**. After deprotection of the Boc protecting group in acidic conditions, the crude product was coupled with the linker (compound **5**) to introduce a Michael acceptor for the irreversible binding affinity. Coupling of the crude Boc-deprotected compound **6** with BODIPY-FL-NHS finalized the synthetic steps to produce Ibrutinib-BFL (**7**) at an overall yield of ~11%.

**Figure 2:**
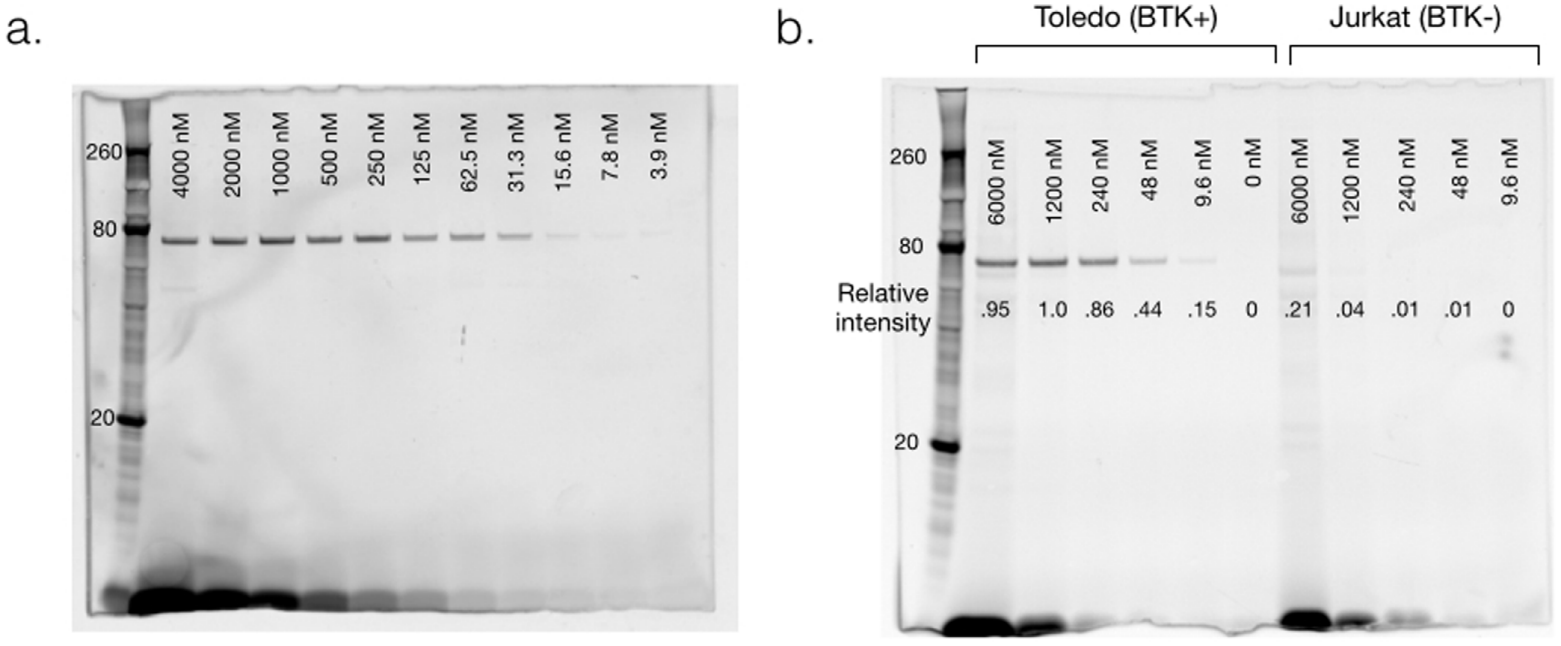
Characterization. a. Target binding. Denaturing gel electrophoresis of decreasing concentrations of Ibrutinib-BFL incubated with 0.1 µg purified BTK for one hour, imaged with 488 nm excitation/520 nm emission. Note the dose dependent binding of Ibrutinib-BFL. Size marker on the far left. b. Denaturing gel electrophoresis of cell lysates following incubation of decreasing concentrations of Ibrutinib-BFL with Toledo (BTK+, left half of gel) or Jurkat (BTK-, right half of gel) cells at 37°C for two hours. Note the superb specificity of the probe.

To confirm the effect of BFL modification on the inhibition efficacy of the drug, half-maximal inhibitory concentration (IC_50_) of Ibrutinib and Ibrutinib-BFL were determined against purified BTK enzyme. Ibrutinib-BFL had an IC_50_ of ~200 nM, which is less potent than the parent drug (~2 nM IC_50_; data not shown). Although it may be possible to further optimize the affinity of Ibrutinib-BFL by testing various linkers, we found the current generation probe to be quite acceptable for imaging, as shown in subsequent experiments. We next determined whether Ibrutinib-BFL would bind to purified BTK in vitro, endogenous BTK in live cells, and ultimately *in vivo*. Purified BTK was incubated with varying concentrations of the imaging probe for one hour at room temperature, denatured at 70°C for 10 minutes and then processed for SDS-PAGE gel analysis. There was a clear dose-response increase of the fluorescent signal around 80 kDa (BTK molecular weight is 76 kDa), as well as at the bottom of the gel (unbound fraction of Ibrutinib-BFL) (Fig. 2a). Additionally, binding could be blocked by pre-incubation with the parent compound and silver staining of the gel showed equal loading of BTK protein (Supplementary Fig. S1). These results clearly confirmed the covalent binding property of Ibrutinib-BFL toward purified BTK.

We next performed a similar experiment in lymphoma cells. We first determined BTK expression in several lymphoma cell lines (Daudi Burkitt's Lymphoma line, and DB, Toledo, and RC-K8 Diffuse Large B-Cell Lymphoma (DLBCL) lines) and one T-cell leukemia line (Jurkat) by Western blot (Supplementary Fig. S1). As expected, T cells did not express BTK. We found high BTK expression in Daudi and Toledo cell lines, and henceforth used Toledo as model BTK-positive cells and Jurkat as negative control cells. Toledo and Jurkat cells were incubated with different doses of Ibrutinib-BFL, and cell lysates were processed for SDS-PAGE and analyzed by fluorescent gel scanning. The imaging probe showed remarkable specificity, with binding observed only at a single band (Fig. 2b). The specificity was further confirmed by the absence of a band in BTK-negative Jurkat cells, even at the highest concentration of probe (Fig. 2b), as well as by silver staining of the gel (Supplementary Fig. S1).

**Figure 3:**
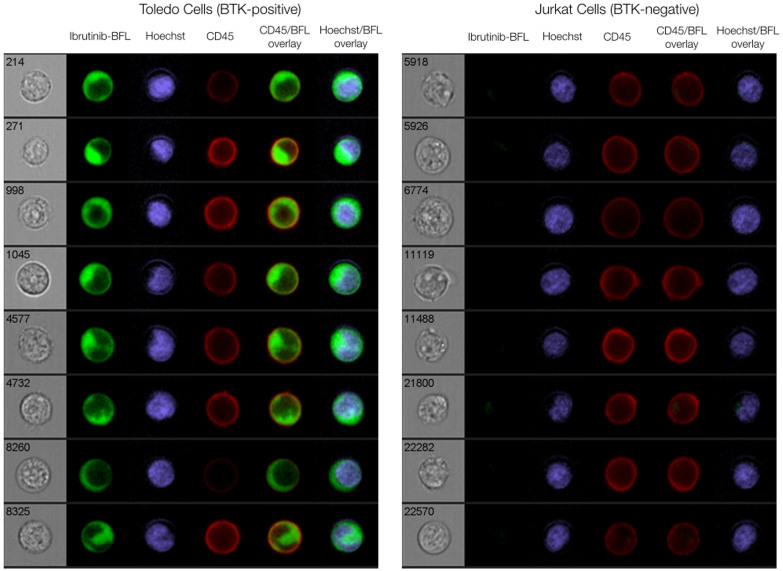
Cellular imaging of lymphoma cells. Representative images of Toledo (BTK-positive; left) and Jurkat (BTK-negative; right) cells incubated with 100 nM Ibrutinib-BFL at 37°C for 2 hours, then in probe-free media at 37°C for 24 hours. Cells were co-stained with Hoechst (nucleus) and CD45 (cell membrane) to show Ibrutinib-BFL localization in the cytoplasm of BTK-positive cells. Note the specificity. Images were obtained with an Amnis ImageStream flow cytometry system.

**Figure 4:**
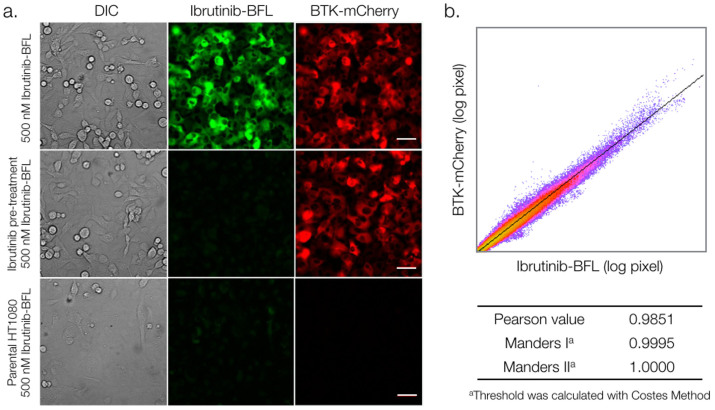
Imaging of adherent BTK-mCherry cells to determine co-localization with Ibrutinib-BFL. a. Imaging co-localization between 500 nM Ibrutinib-BFL (green) and HT1080 cells stably transfected with BTK-mCherry (red), following a 2-hour incubation with Ibrutinib-BFL and then a 24-hour incubation in probe-free media (top). Center: competitive inhibition with 1 µM Ibrutinib prior to Ibrutinib-BFL addition. Bottom: Ibrutinib-BFL incubated with non-BTK expressing parent HT1080 cells. b. Note the exquisite co-localization. Scale bar: 50 µm.

We next performed live cell imaging experiments using an imaging flow cytometry system. To prepare Toledo and Jurkat cells, we incubated them with 100 nM Ibrutinib-BFL for two hours, followed by washing. Figure 3 and Supplementary Fig. S2 summarize some of the results confirming target binding, specificity via blocking, and the ability to perform live cell imaging. To quantify co-localization between the imaging probe and BTK at the subcellular level, we created a stable transgenic cell line expressing a BTK-mCherry fusion protein in HT1080 human fibrosarcoma cells. *In vitro* cell experiments showed excellent co-localization and blocking (r^2^ = 0.9851; Fig. 4).

**Figure 5:**
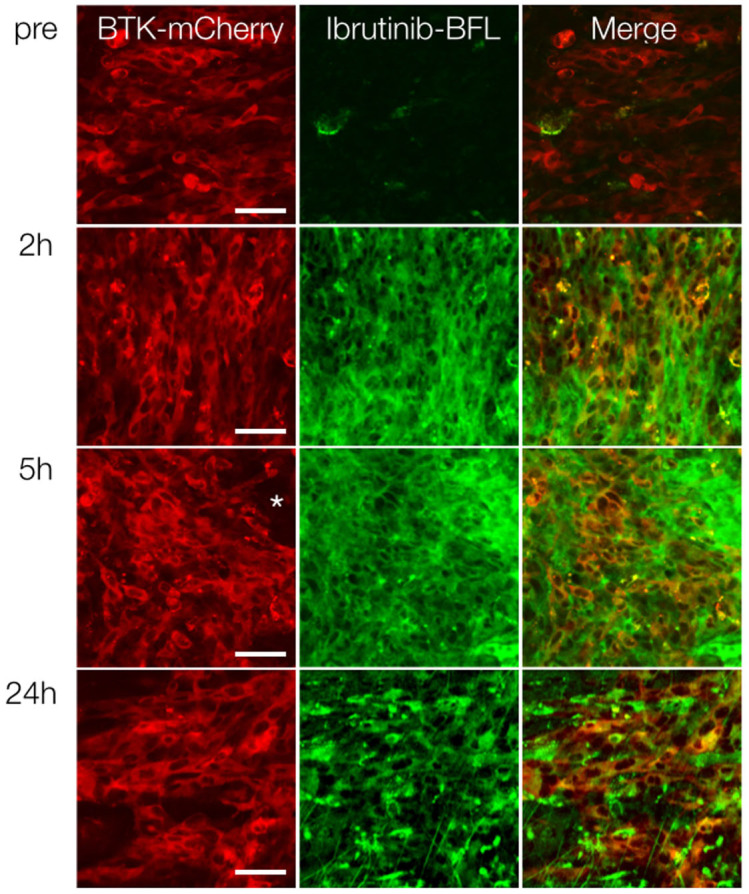
In vivo tumor imaging. Serial imaging before, and at 2, 5 and 24 hours after intravenous administration of Ibrutinib-BFL to a representative mouse harboring a BTK-positive HT1080 tumor (red; first column). Note extensive drug accumulation in all cells, persisting even at the 24-hour time point. * Indicates accumulation in non-tumor cells (see Fig. 6). Scale bar: 50 µm.

**Figure 6:**
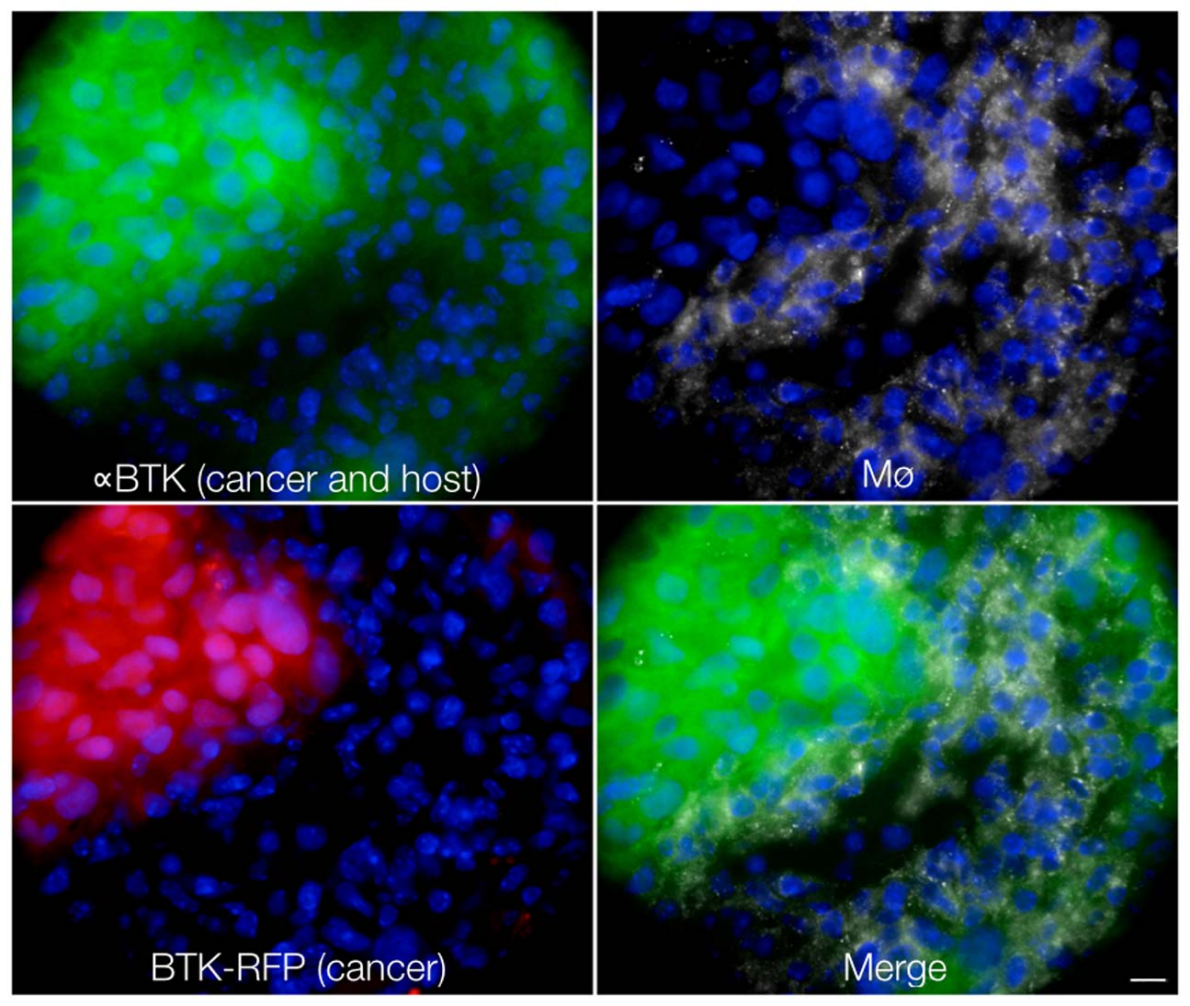
Histology. To corroborate intravital serial imaging, tumors were examined histologically. Anti-BTK staining showed BTK signal in HT-1080-BTK-mCherry cells as expected, but also in tumor-associated macrophages (white). These regions of drug accumulation correspond to those seen by intravital imaging (* in Fig. 5). Scale bar: 10 µm.

We next performed *in vivo* experiments using three-color (blue: vasculature, green: Ibrutinib-BFL, red: BTK-mCherry-HT1080 cells) time-lapse intravital imaging. The intravascular half-life of Ibrutinib-BFL was ~10 minutes (Supplementary Fig. S3). Within an hour after systemic administration, there was extensive leakage of the compound into the tumor interstitium. At later time points, cellular uptake became apparent, presumably due to interstitial washout and/or intracellular accumulation. The ability to image in multiple channels allowed us to ask whether Ibrutinib specifically localized in tumor cells. We show that greater than 99% of all BTK-mCherry-HT1080 cells had achieved therapeutic drug concentrations within one hour. This effective intracellular dose persisted for prolonged periods of time and the compound was still detectable inside cancer cells 24 hours after administration (Fig. 5). Interestingly, there was also accumulation of Ibrutinib-BFL in non-tumor cells even at late time points. Given the exquisite specificity of the drug (see Fig. 2), we hypothesized that these non-target cells also contain BTK. We thus performed correlative immunohistochemistry using anti-BTK antibody. Our data indicates that Ibrutinib-BTK also accumulates in tumor-associated macrophages and lymphocytes (Fig. 6).

## Discussion

Inhibition of BTK is emerging as a promising target for B-cell malignancies, other cancers with BTK over-expression, and certain autoimmune diseases where BTK is involved. Ibrutinib, an irreversible inhibitor, is approved for treatment of mantle cell lymphoma and CLL, and is currently undergoing late-stage efficacy studies in patients with various B-cell malignancies. Based on its covalent target binding, we hypothesized that the molecule could serve as a companion imaging agent. Here we show that this is indeed the case. Ibrutinib-BFL co-localized with BTK in BTK-positive malignant cells and had low background accumulation in non-BTK cells, including those expressing structurally related interleukin-2-inducible T-cell kinase (ITK), which is expressed in T cells and Jurkat cells (see Supplementary Fig. S1). The companion imaging drug, Ibrutinib-BFL, also showed a predictable dose response curve, could be competitively inhibited, allowed drug concentrations to be quantitated *in vivo*, and enabled mapping of drug distributions at the single cell level. As such, we believe that Ibrutinib-BFL could have several applications, including use as a companion diagnostic for flow cytometry in haematologic malignancies, as an imaging agent to localize and map BTK-positive tumors, as a method to track subcellular localization of endogenous BTK, and as a tool to measure pharmacokinetics and pharmacodynamics in experimental settings during development of novel BTK-pathway inhibitors.

BTK is a cytoplasmic tyrosine kinase belonging to the Tec family. It is expressed in the B-cell lineage, plays a pivotal role in signaling and development, and is highly active in several haematological malignancies^28^,^29^. Some previous BTK imaging has been done with fluorescent protein tags (BTK-GFP and BTK-mCherry) to understand its activation and nucleocytoplasmic shuttling^30^,^31^,^32^, and its roles in myeloid cell chemotaxis^33^ and infection^34^,^35^. Alternative research methods have primarily involved fluorescently labeled antibodies for immunohistochemistry and flow cytometry applications. The former is limited to experimental models and requires protein over-expression, and the latter requires cell permeabilization and fixation. The approach developed here, utilizing a small molecule affinity ligand, is compatible with live cells, can be used *in vivo*, and has potential clinical applicability. Not only does Ibrutinib-BFL specifically bind to BTK, but also it remains bound until protein turnover due to the virtually nonexistent off-rate of covalent inhibitors. This feature will allow for long-term study of endogenous BTK in live cells, providing a window into drug pharmacodynamics, as well as innate heterogeneity in responses to drugs targeting the BCR signaling pathway^24^,^27^.

Beyond utilizing Ibrutinib-BFL in pharmacologic studies of next generation inhibitors, there are future diagnostic opportunities in which BTK-expressing lymphomas could be imaged in the clinic. While the current work focused on single cell imaging *in vivo*, we also anticipate whole body imaging applications. For example, the fluorine in BODIPY-FL could be exchanged for ^18^F for positron emission tomography (PET) imaging, or entirely replaced via bioorthogonal ligands or direct ^18^F attachment^36^,^37^,^38^,^39^. Alternatively, longer-lived isotopes such as Zirconium-89 could also be utilized in order to take full advantage of the probe's irreversible binding kinetics^40^,^41^,^42^,^43^,^44^. Such molecules may be useful in clinical imaging-based tests for whole body distribution and inhibition of BTK. Other areas of interest are to use these molecules for imaging BTK in macrophages during infection, or to use them as a readout during gene therapy for the immunodeficiency disorder X-linked agammaglobulinemia, which results from loss of functional BTK^45^. Irrespective of the contemplated use, we believe that the developed agent should be useful in a number of different applications. As covalent inhibitors have gained interest, we anticipate covalent imaging agents to follow, and Ibrutinib-BFL can provide a roadmap for such development.

## Methods

### Synthesis and Characterization of Probe

All reagents were obtained from commercial sources and used without further purification. Dry THF, MeOH, DCM, and DMF were obtained from Sigma-Aldrich (St. Louis, MO). ^1^H and ^13^C NMR spectra were recorded at 23°C on a Bruker 400 MHz spectrometer. Recorded shifts are reported in parts per million (δ) and calibrated using residual undeuterated solvent. Data are represented as follows: chemical shift, multiplicity (s = singlet, d = doublet, t = triplet, q = quartet, p = pentet, m = multiplet, br = broad), coupling constant (*J*, Hz), and integration. LC-ESI-MS analysis and HPLC-purifications were performed on a Waters (Milford, MA) LC-MS system. For LC-ESI-MS analyses, a Waters XTerra® C18 5 μm column was used. For preparative runs, an Atlantis® Prep T3 OBDTM 5 μm column was used [eluents 0.1% TFA (v/v) in water (solution A) and MeCN (solution B); gradient: 0–1.5 min, 5–100% B; 1.5–2.0 min, 100% B for analysis and 0–0.75 min, 5% B; 0.75–9.0 min, 5–100% B; 9.0–10.0 min, 100% B for prep.].

### 3-iodo-1H-pyrazolo[3,4-d]pyrimidin-4-amine (1)

A solution of 4-amino-1H-pyrazolo[3,4-d]pyrimidine (780 mg, 5.77 mmol) and N-Iodo-succinimide (2.02 g, 8.98 mmol) in DMF (6 mL) was stirred at 80°C overnight. Resulting brown solution was filtered and sticky solid was washed with water and cold ethanol. Resulting light yellow solid was dried in vacuo to give compound **1** (1.50 g, 99.6% yield). Crude product was used for the next reaction without further characterization.

### 3-(4-phenoxyphenyl)-1H-pyrazolo[3,4-d]pyrimidin-4-amine (2)

A solution of compound **1** (200 mg, 0.77 mmol), tetrakis-(triphenylphosphine)palladium (124 mg, 0.11 mmol), potassium phosphate tribasic (488 mg, 2.3 mmol), and 4-phenoxyphenylboronic acid (492 mg, 2.3 mmol) in 1,4-dioxane (2.5 mL) in a microwave vial was heated to 180°C for 10 minutes under microwave irradiation. Resulting reaction mixture was diluted with water and organic material was extracted with EA three times. Combined organic material was dried over Na_2_SO_4_ and concentrated in vacuo. Resulting yellow solution was dissolved with DCM and resulting turbid solution was filtered to give compound **2** as a white solid (138 mg, 59.4% yield).^1^H NMR (400 MHz, DMSO) δ 13.55 (s, 1H), 8.24 (s, 1H), 7.67 (d, *J* = 8.2 Hz, 2H), 7.44 (t, *J* = 7.8 Hz, 2H), 7.17 (m, 5H); ^13^C NMR (101 MHz, DMSO) δ 158.0, 157.0, 156.3, 156.0, 155.7, 143.9, 130.1, 130.0, 128.4, 123.7, 119.0, 118.9, 96.9; LRMS (ESI) *m*/*z* calcd for C_17_H_13_N_5_O [M+H]^+^ 304.12, found 304.14.

### (R)-*tert*-butyl 3-(4-amino-3-(4-phenoxyphenyl)-1H-pyrazolo[3,4-d]pyrimidin-1-yl)piperidine-1-carboxylate (3)

A solution of compound **2** (57 mg, 0.19 mmol), (S)-3-hydroxy-N-Boc-piperidine (80 mg, 0.40 mmol), DIAD (150 µL, 0.764 mmol), and polymer-TPP (0.5 g, 1.6 mmol) in THF (4 mL) was stirred at ambient temperature overnight. After reaction completion, polymer-TPP was removed by filtration, filtrate was concentrated in vacuo and purified with silica gel column chromatography (EA : Hex = 0 : 100 to EA only) to give compound **3** (45 mg, 48.7% yield) as a clear oil.^1^H NMR (400 MHz, CDCl_3_) δ 8.37 (s, 1H), 7.65 (d, *J* = 7.5 Hz, 2H), 7.38 (m, 2H), 7.19 – 7.12 (m, 3H), 7.08 (d, *J* = 8.6 Hz, 2H), 5.49 (s, 2H), 4.84 (dq, *J* = 10.4, 5.1, 4.3 Hz, 1H), 4.35 – 4.23 (m, 1H), 4.16 – 4.06 (m, 1H), 3.46 (t, *J* = 11.5 Hz, 1H), 2.88 (td, *J* = 12.3, 2.8 Hz, 1H), 2.34 – 2.14 (m, 2H), 1.96 – 1.85 (m, 1H), 1.78 – 1.65 (m, 1H), 1.45 (s, 9H). ^13^C NMR (101 MHz, CDCl_3_) δ 158.5, 157.8, 156.4, 155.7, 154.6, 154.2, 143.6, 130.0, 129.9, 127.9, 124.0, 119.5, 119.1, 98.6, 79.8, 52.9, 48.2, 44.0, 30.2, 28.4, 24.5. LRMS (ESI) *m*/*z* calcd for C_27_H_30_N_6_O_3_ [M+H]^+^ 487.24, found 487.25.

### (E)-ethyl 4-((tert-butoxycarbonyl)amino)but-2-enoate (4)

To a solution of NaH (50 mg, 1.26 mmol) in THF (4 mL), stirred at 0°C, triethylphosphonoacetate (374 µL) was added dropwise. After warming up to ambient temperature, solution of N-Boc-2-aminoacetaldehyde (100 mg, 0.63 mmol) in THF (1 mL) was added. Reaction mixture was stirred at ambient temperature. After reaction completion, reaction mixture was diluted with water and organic material was extracted with EA three times. Combined organic material was dried over Na_2_SO_4_ and concentrated in vacuo. Reaction mixture was purified with silica gel column chromatography (EA : Hex = 0 : 100 to EA only) to give compound **4** (110 mg, 76.4% yield) as a clear oil. ^1^H NMR (400 MHz, CDCl_3_) δ 6.88 (dt, *J* = 15.7, 4.9 Hz, 1H), 5.91 (dt, *J* = 15.6, 1.8 Hz, 1H), 4.81 (s, 1H), 4.16 (q, *J* = 7.2 Hz, 2H), 3.89 (s, 2H), 1.42 (s, 9H), 1.26 (t, *J* = 7.1 Hz, 3H); ^13^C NMR (101 MHz, CDCl_3_) δ 166.2, 155.7, 144.9, 121.4, 79.9, 60.5, 41.4, 28.4, 14.3; LRMS (ESI) *m*/*z* calcd for C_11_H_19_NO_4_ [M+H]^+^ 230.13, found 230.15.

### (E)-4-((tert-butoxycarbonyl)amino)but-2-enoic acid (5)

A solution of compound **4** (110 mg, 0.48 mmol) and LiOH (168 mg, 2.4 mmol) in THF (3 mL) and water (2 mL) was stirred at ambient temperature overnight. THF was evaporated and resulting yellow aqueous solution was acidified with 1N HCl to pH 3. Organic material was extracted with DCM three times. Combined organic material was dried over Na_2_SO_4_ and concentrated in vacuo. Reaction mixture was purified with silica gel column chromatography (MeOH : DCM = 0 : 100 to 1 : 10) to give compound **5** (80 mg, 82.9% yield) as a white solid.^1^H NMR (400 MHz, CDCl_3_) δ 6.93 (dt, *J* = 15.9, 4.7 Hz, 1H), 5.87 (dt, *J* = 15.7, 1.9 Hz, 1H), 4.73 (s, 1H), 3.88 (s, 2H), 1.39 (s, 9H); ^13^C NMR (101 MHz, CDCl_3_) δ 170.9, 155.8, 147.4, 120.8, 80.2, 41.5, 28.5; LRMS (ESI) *m*/*z* calcd for C_9_H_15_NO_4_ [M+H]^+^ 202.10, found 202.10.

### (R,E)-tert-butyl (4-(3-(4-amino-3-(4-phenoxyphenyl)-1H-pyrazolo[3,4-d]pyrimidin-1-yl)piperidin-1-yl)-4-oxobut-2-en-1-yl)carbamate (6)

A solution of compound **3** (100 mg, 0.21 mmol) in 2 mL of TFA and DCM mixture (1 : 3 = v : v) was stirred at ambient temperature. After 30 minutes stirring, reaction mixture was concentrated in vacuo. After azeotropic distillation with DCM and ACN three times, crude product was concentrated in vacuo. Then crude product was diluted with DMF (2.1 mL) to make 0.1 M solution. 650 µL of crude product solution in DMF was mixed with compound **5** (16 mg, 0.078 mmol), HBTU (37 mg, 0.097 mmol), and TEA (45 µL) in DMF (200 µL) and reaction mixture was stirred at ambient temperature. After one hour, reaction mixture was directly loaded onto a C18 reverse phase column for purification (Water : ACN w/0.1% Formic acid = 95 : 5 to 0 : 100) to give compound **6** (25 mg, 67.8% yield) as a sticky solid.^1^H NMR (400 MHz, CDCl_3_) δ 8.36 (d, 1H), 7.72 – 7.57 (m, 3H), 7.38 (t, *J* = 7.8 Hz, 2H), 7.21 – 7.02 (m, 5H), 6.65 (t, *J* = 9.8 Hz, 1H), 5.75 (br s, 2H), 4.94 – 4.74 (m, 1H), 4.74 – 4.60 (m, 1H), 4.52 (d, *J* = 13.0 Hz, 0.5H), 4.10 – 3.97 (m, 0.5H), 3.86 (d, *J* = 13.2 Hz, 0.5H), 3.66 (dd, *J* = 13.2, 10.4 Hz, 0.5H), 3.33 (t, *J* = 12.0 Hz, 0.5H), 3.26 – 2.95 (m, 2H), 2.85 – 2.66 (m, 0.5H), 2.50 – 2.15 (m, 2H), 2.06 – 1.86 (m, 1H), 1.80 – 1.58 (m, 1H), 1.46 (s, 9H); LRMS (ESI) *m*/*z* calcd for C_31_H_35_N_7_O_4_ [M+H]^+^ 570.28, found 570.20.

### (R,E)-3-(3-((4-(3-(4-amino-3-(4-phenoxyphenyl)-1H-pyrazolo[3,4-d]pyrimidin-1-yl)piperidin-1-yl)-4-oxobut-2-en-1-yl)amino)-3-oxopropyl)-5,5-difluoro-7,9-dimethyl-5H-dipyrrolo[1,2-c:2′,1′-f][1,3,2]diazaborinin-4-ium-5-uide (7)

A solution of compound **6** (5 mg, 0.009 mmol) in 2 mL of TFA and DCM mixture (1 : 3 = v : v) was stirred at ambient temperature. After 30 minutes stirring, reaction mixture was concentrated in vacuo. After azeotropic distillation with DCM and ACN for three times, crude product was concentrated in vacuo. Then crude product was diluted with DMF (880 µL) to make 0.1 M solution. 390 µL of crude product solution in DMF was mixed with BODIPY-FL-NHS (1 mg, 0.0026 mmol) and TEA (2 µL, 0.013 mmol) in DMSO (1 mL) and the resulting reaction mixture was stirred at ambient temperature for one hour and was then purified using standard HPLC techniques to give compound **7** (1.1 mg, 57.6% yield) as a greenish solid.^1^H NMR (400 MHz, CDCl_3_) δ 8.45 (br s, 1H), 8.23 (d, *J* = 9.9 Hz, 1H), 7.63 (dd, *J* = 32.3, 8.2 Hz, 2H), 7.48 - 7.21 (m, 3H), 7.21 - 7.06 (m, 2H), 7.04 - 6.86 (m, 2H), 6.66 (dt, *J* = 4.9, 4.9, 15.3 Hz, 1H), 6.52 - 6.28 (m, 2H), 6.18 (d, *J* = 22.1 Hz, 1H), 4.56 (d, *J* = 12.3 Hz, 1H), 4.24 (dd, *J* = 13.3, 39.5 Hz, 2H), 4.03 - 3.78 (m, 3H), 3.54 - 3.44 (m, 1H), 3.27 - 3.07 (m, 3H), 2.74 - 2.62 (m, 1H), 2.60 - 2.30 (m, 5H), 2.29 - 2.17 (m, 3H), 2.09 - 1.99 (m, 1H), 1.74 - 1.60 (m, 1H); LRMS (ESI) *m*/*z* calcd for C_40_H_40_BF_2_N_9_O_3_ [M+H]^+^ 744.34, found 744.30.

### Cell Lines

The diffuse large B-cell lymphoma (DLBCL) cell lines DB and Toledo were generously provided by Dr. Anthony Letai (Dana Farber Cancer Institute, Boston, MA, USA). The RC-K8 DLBCL cell line was a generous gift from Dr. Thomas Gilmore (Boston University, Boston, MA, USA). Daudi Burkitt's lymphoma cell line and Jurkat T-cell leukemia line were from ATCC (Manassas, VA, USA). Lymphoma cell lines were cultured in RPMI 1640 media supplemented with 10% fetal bovine serum at 37°C and 5% CO_2_. To test the BTK inhibitor in adherent cells, we used HT1080 human fibrosarcoma cells, which have previously been shown to be ideal for intravital imaging studies^46^. HT1080 cells were from ATCC, grown in DMEM supplemented with 10% fetal bovine serum and 2% glutamine-penicillin-streptomycin at 37°C and 5% CO_2_. HT1080-BTK-mCherry cells were prepared by viral infection of HT1080 cells. Virus generated from pMSCVpuro-mCherry-BTK retroviral vector^35^ was a generous gift from Dr. Hidde Ploegh (Massachusetts Institute of Technology, Cambridge, MA, USA). Viral supernatant was added directly to HT1080 cells for 48 hours, and BTK-mCherry-expressing cells were then selected with RPMI media containing 2 µg/mL puromycin for 96 hours. Following selection, HT1080-BTK-mCherry cells were cultured under the same conditions as the original HT1080 cells.

### Gel Electrophoresis

To test the covalent binding of Ibrutinib-BFL to BTK, 0.1 µg (1 µL) purified BTK was combined with 0.4 µL Ibrutinib-BFL (prepared in advance in 2-fold dilutions ranging from 200 µM to 0.19 µM, 33% DMSO in PBS) and 18.6 µL PBS, and incubated in the dark at room temperature for one hour. In the second experiment, Toledo and Jurkat cells (2.2 × 10^6^ per well in culture media) were incubated in growth media containing 5-fold serial dilutions of Ibrutinib-BFL ranging from 6 µM to 9.6 nM in final 2% DMSO at 37°C for two hours. Control samples were incubated in growth media containing 2% DMSO. Cells were washed once with ice cold PBS, then lysed in 150 µL 1X RIPA buffer (Cell Signaling Technology, Beverly, MA, USA) containing protease inhibitors. To the purified enzyme samples or cell lysates, NuPAGE LDS sample buffer and NuPAGE reducing agent (Invitrogen) were added for final 25% and 10% concentrations, respectively, and samples were heated to 70°C for 10–12 minutes in a Mastercycler thermal cycler (Eppendorf, Hamburg, Germany). 25 µL per lane was loaded into 12-well NuPAGE Novex 4–12% Bis-Tris gels (Invitrogen). Using 10 µL of Novex Sharp Pre-stained Protein Standard (Invitrogen) as a size marker, the gels were run in NuPAGE MES SDS running buffer (Invitrogen) at 200 V for 35 minutes in the XCell SureLock Mini-Electrophoresis system (Invitrogen). The gels were removed from the cassette and imaged using a Typhoon 9410 fluorescence scanner (GE Healthcare, Pittsburgh, PA, USA) using 488 nm excitation and a 520 nm emission filter. To show total protein loading, gels were silver-stained using the Pierce Silver Stain for Mass Spectrometry kit (Thermo Fisher Scientific, Rockford, IL, USA).

### Imaging of non-adherent lymphoma cells by flow cytometry

Jurkat and Toledo cells were single- or triple-stained with the following, followed by washing: Ibrutinib-BFL (2 hours in growth media, 37°C), Hoechst 33342 nuclear dye (Invitrogen), or APC-conjugated anti-human-CD45 antibody (Clone HI30, BioLegend, San Diego, CA, USA)(both 30 minutes in PBS containing 2% BSA, 4°C). Stained cells were transferred to Clear-view Snap-Cap microtubes (Sigma-Aldich) for Amnis ImageStream^X^ Mark II imaging flow cytometry (Amnis Corporation, Seattle, WA, USA). Single-stained samples were used to create a compensation table, then 30,000 images from each triple-stained sample were collected using excitation lasers 405-nm, 488-nm, 592-nm, and bright-field excitation, and 430-550-nm (Ch7), 480-560-nm (Ch2), 640–745-nm (Ch11), and 430–480 (Ch1) emission filters. Representative images were manually selected from this data set.

### Imaging of adherent cells by microscopy

HT1080-BTK-mCherry cells were seeded into a 96-well plate at 20,000 cells per well and allowed to grow to confluence overnight. Cells were incubated in growth media containing 1 µM Ibrutinib in final 0.1% DMSO, or control 0.1% DMSO, at 37°C for 1.5 hours. Without washout, a 50× stock of Ibrutinib-BFL in 5% DMSO was added for a final concentration of 500 nM. Control wells contained equivalent DMSO without Ibrutinib-BFL. Cells were incubated for one hour at 37°C and then washed once with media for five minutes. The media was then replaced and cells were incubated overnight at 37°C. The live cells were subsequently imaged on the DeltaVision imaging system (Applied Precision, a GE Healthcare Company). Images were processed with Fiji software, an open-source version of ImageJ.

### In vivo tumor imaging

Nu/nu mice were implanted with 2 × 10^6^ HT1080-BTK-mCherry cells into a dorsal skinfold window chamber (APJ Trading Company, Ventura, CA, USA) according to established protocols^47^ and according with guidelines from the Institutional Subcommittee on Research Animal Care. Tumors were allowed to grow and vascularize for two weeks. 75 nmol Ibrutinib-BFL in 150 µL solution containing DMAc and solutol was injected via tail vein as reported previously^48^. Mice were anesthetized with 2% isoflurane in 2 L/min oxygen. Time-lapse microscopy was performed for two hours using a customized Olympus FV1000 confocal/multiphoton microscope equipped with a 20× objective (both Olympus America, Chelmsford, MA, USA). In addition, tumors were imaged before injection, and at 2, 5, and 24 hours post-injection. Images were processed with Fiji software.

### Histology

HT1080-BTK-mCherry tumors were harvested from nu/nu mice and embedded in O.C.T. compound (Sakura Finetek, Torrance, CA, USA). Serial 6 μm-thick frozen sections were prepared for histological analysis. Fluorescence immunohistochemistry staining was performed using Mac-3 (clone: M3/84, BD Biosciences, San Jose, CA, USA) and BTK (clone: D3H5, Cell Signaling Technology), followed by Alexa Fluor 647 goat anti-rat IgG and Alexa Fluor 488 goat anti-rabbit IgG (both Invitrogen) secondary antibodies, respectively. Images were captured using a BX63 fluorescence microscope (Olympus America) equipped with a Neo sCMOS camera (Andor Technology, Belfast, UK) and processed with Fiji software.

## Author Contributions

A.T., E.K. and R.W. designed the research and A.T., E.K. performed experiments. E.K. performed chemical synthesis and characterization. M.A.M. prepared transgenic cell lines and provided guidance with animal models. R.H.K. performed intravital imaging. A.T., E.K., R.H.K. and R.W. prepared figures. A.T., E.K. and R.W. wrote the manuscript. All authors reviewed and edited the manuscript.

## Supplementary Material

Supplementary Information SUPPLEMENTARY INFORMATION
