# Supplementary material for: Single cell imaging of Bruton's Tyrosine Kinase using an irreversible inhibitor
Source: Sci Rep. 2014 Apr 24;4:4782. doi: 10.1038/srep04782 (PMC3998017; doi:10.1038/srep04782)

SUPPLEMENTARY INFORMATION

Single cell imaging of Bruton's Tyrosine Kinase using an irreversible inhibitor

Anna Turetsky[1,a], Eunha Kim[1,a], Rainer H. Kohler[1], Miles A. Miller[1], Ralph Weissleder[1,2,]*

[1] Center for Systems Biology, Massachusetts General Hospital, 185 Cambridge St, CPZN 5206, Boston, MA 02114

[2] Department of Systems Biology, Harvard Medical School, 200 Longwood Ave, Boston, MA 02115

[a] equal contribution

Supplementary Methods

Kinase Inhibition Assay

The Z'-LYTE Kinase Assay Kit - Tyrosine 1 Peptide (Invitrogen, Grand Island, NY, USA) was used according to the manufacturer's instructions and tested against purified BTK enzyme (Promega, Madison, WI, USA) and ATP. The assay was then performed in the presence of inhibitors Ibrutinib and Ibrutinib-BFL using 1 µg/mL BTK and 50 µM ATP, without pre-incubation of inhibitors with the enzyme. Concentrations of Ibrutinib were 4-fold serial dilutions ranging from 33 nM to 0.01 nM, while concentrations of Ibrutinib-BFL were 4-fold serial dilutions ranging from 8000 nM to 1.95 nM. Negative control samples did not contain ATP, and positive controls contained the pre-phosphorylated peptide included in the assay kit. Kinase reaction buffer contained 0.67% DMSO. Experiments were performed in quadruplicate in 384-well plates. Fluorescence resulting from peptide phosphorylation was measured on a TECAN plate reader system (Zurich, Switzerland). Background fluorescence from the fluorescent probe was subtracted from the final measurements. Prism (GraphPad Software, La Jolla, CA, USA) was used to plot dose-response curves and to calculate IC50 values.

Western Blot

Cell lysates were prepared from B-cell lymphoma (Daudi, DB, Toledo, RC-K8) and control T cell (Jurkat) lines by lysis in RIPA buffer, and total protein concentrations were measured using the TECAN plate reader after performing the Pierce BCA protein assay (Thermo Fisher Scientific) according to the manufacturer's instructions in a 96-well plate format. 17.5 µg of each cell line was diluted with H2O to 20 µL, and samples were then combined with NuPAGE LDS sample buffer and NuPAGE reducing agent (Invitrogen) for final 25% and 10% concentrations, respectively, and samples were heated to 70°C for 10 minutes. Samples were loaded onto a 12-well NuPAGE Novex 4-12% Bis-Tris Gel (Invitrogen). Using Novex Sharp Pre-stained Protein Standard (Invitrogen) as a size marker, the gel was run in MES buffer at 200 V for 35 minutes. An iBlot blotting system (Invitrogen) was used for transfer to a PVDF membrane. Membranes were blocked in Pierce SuperBlock blocking buffer (Thermo Fisher Scientific) for one hour, then stained overnight at 4°C in 1:500 mouse anti-human-BTK antibody (Clone 53/BTK, BD, Franklin Lakes, NJ, USA) in TBST buffer containing 10% SuperBlock. Blots were then washed in TBST three times for 20 minutes and incubated in 1:1000 Peroxidase Affinipure goat anti-mouse IgG (Jackson ImmunoResearch Laboratories, West Grove, PA, USA) in TBST buffer containing 10% SuperBlock for one hour at room temperature. After washing three times for 20 minutes each, blots were incubated with SuperSignal West Pico Chemiluminescent Substrate (Thermo Fisher Scientific), exposed on film, and developed with a Kodak X-OMAT 2000A processor (Rochester, NY, USA).

Flow Cytometry

Jurkat and Toledo cells were incubated in growth media containing 5-fold serial dilutions of Ibrutinib-BFL, ranging from 8 μM to 12.8 nM in final 2% DMSO at 37°C for two hours. Control samples contained cells in media containing 2% DMSO. Cells were centrifuged and resuspended in growth media at time-points of 1, 14, 24, and 72 hours. At each time-point, $5x10^5$ cells were harvested for analysis by flow cytometry, and the remaining cells were cultured at 37°C. 24 hours was found to be the optimal washout time-point and used in subsequent experiments. A separate, blocking experiment was performed in which Toledo cells were first incubated in growth media containing 5-fold serial dilutions of unmodified Ibrutinib (Cellagen Technology, San Diego, CA, USA) ranging from 500 nM to 0.8 nM in final 2% DMSO at 37°C for 1.5 hours. Without washout, Ibrutinib-BFL was then added to each well for 50 nM final concentration and 4% final DMSO, and incubated at 37°C for two hours. A positive control sample contained no Ibrutinib, while a negative control for autofluorescence contained no Ibrutinib-BFL. After two hours, Ibrutinib-BFL-containing media was removed by centrifugation, and cells were incubated in fresh media at 37°C for 24 hours prior to analysis by flow cytometry. Cells collected for flow cytometry were centrifuged and resuspended in 200 μL PBS containing 0.5% BSA, and filtered through 35 μM Cell Strainer tubes (BD). Flow cytometry in the FITC channel was performed on an LSRII flow cytometer (BD) and subsequent data analysis was done using FlowJo software (TreeStar, Ashland, OR, USA).

Supplementary Figures

Supplementary Fig. S1. Gel electrophoresis experiments. *a. Competition*. After pre-incubation of BTK with concentrations of Ibrutinib ranging from 80 to 2.5 nM for one hour, Ibrutinib-BFL is blocked from binding to the BTK. Loading of Ibrutinib-BFL can be seen at the bottom of the gel. Extent of blocking seems to depend more on time than on concentration of Ibrutinib. *b. Western blot* of lymphoma cell lines, blotted with anti-BTK antibody (left) or stimulated and blotted with an antibody with cross-reactivity for pBTK and pITK, expressed in T cells. Orange dotted line indicates 80 kDa size marker; BTK is reported as having molecular weight of 76 kDa. *c. Silver stain* of purified BTK loading in Figure 2a. *d. Silver stain* of cell lysate loading in Figure 2b.

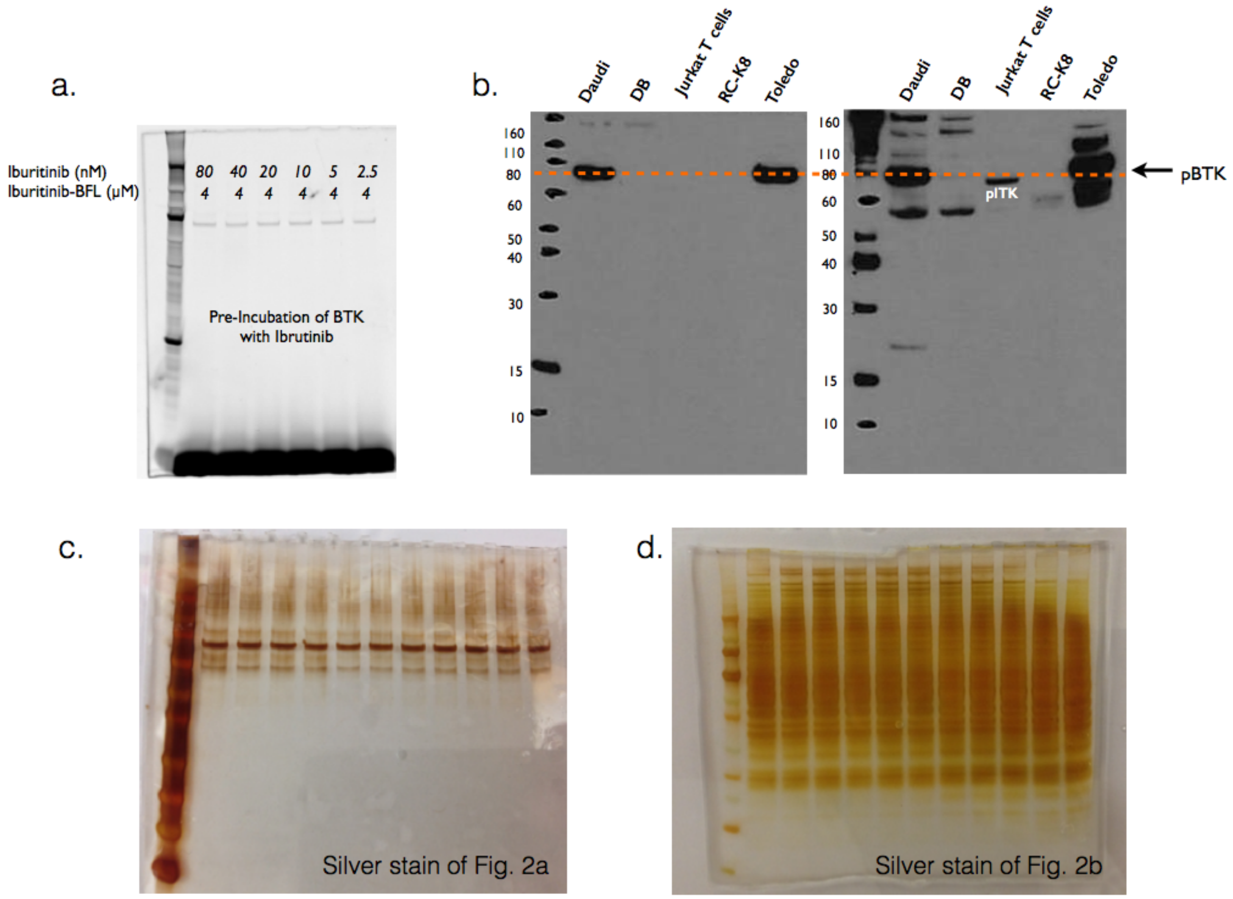

Supplementary Fig. S2. Optimization of BTK imaging and blocking experiment. a. Staining cells with Ibrutinib-BFL. Toledo and Jurkat cells were incubated with 0 nM (top) or 50 nM (bottom) Ibrutinib-BFL for 2 hours, followed by a 24-hour washout period in probe-free media. Ibrutinib-BFL only remains bound to Toledo cells, as shown by clear separation of cell populations by flow cytometry. b. Blocking with Ibrutinib. (Top) Difference in fluorescence between Toledo cells stained with 100 nM Ibrutinib-BFL and unstained Toledo cells. Unstained cells provide a benchmark for 100% blocking (Bottom). Blocking of BTK staining by pre-incubation of Toledo cells with varying concentrations of unmodified Ibrutinib for 1.5 hours, followed by staining with Ibrutinib-BFL. Greater than 90% blocking at all concentrations above the Ibrutinib IC_{50} (~1 nM) confirms that Ibrutinib-BFL binds the same target as Ibrutinib.

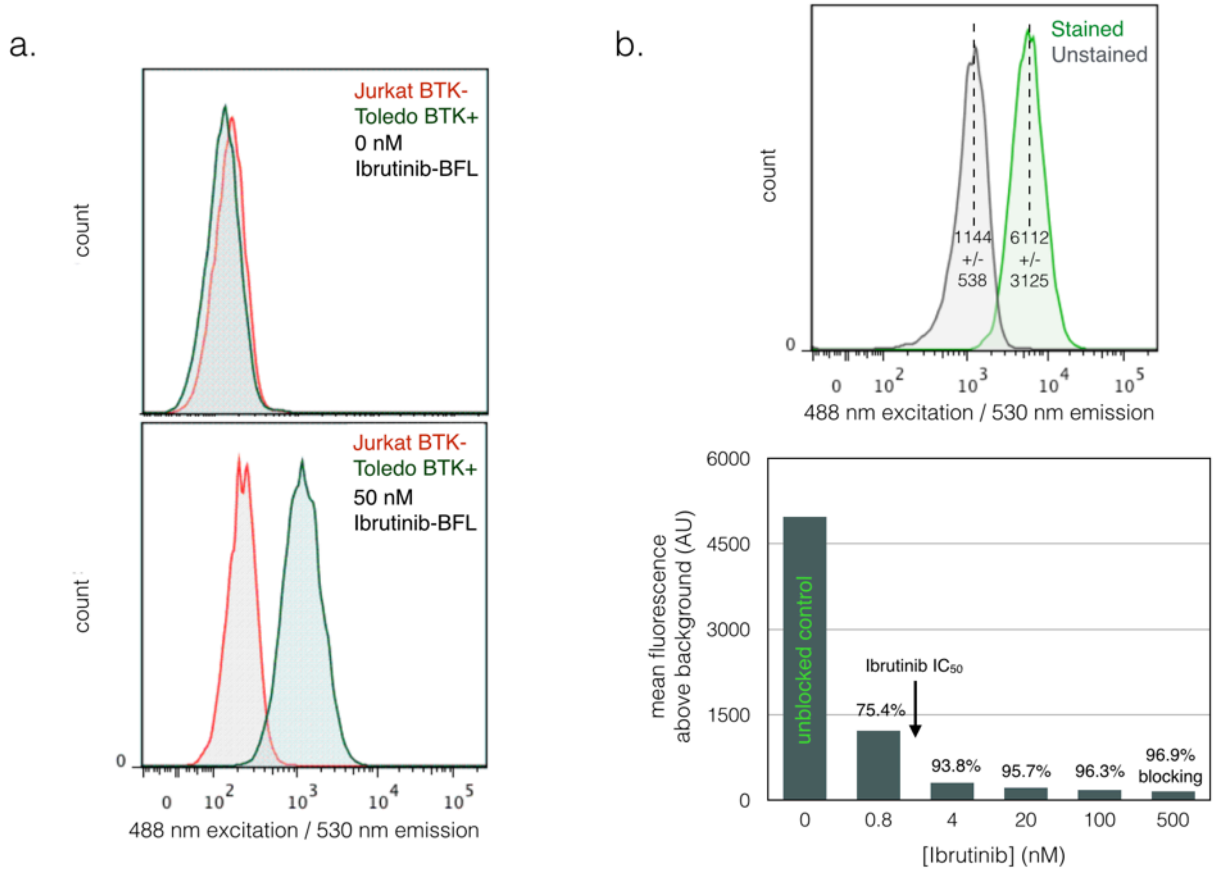

Supplementary Fig. S3. Vascular half-life. 75 nmol Ibrutinib-BFL was injected into the tail vein of C57BL/6 mice and serial imaging was performed in the mouse ear to determine vascular half-life of the IV injected probe. Imaging was performed using a customized Olympus IV110 laser-scanning microscope equipped with a 10x objective. a. Images of vasculature (red) and Ibrutinib-BFL (green) over 60 minutes post injection. b. Fluorescence in the mouse ear vasculature was used to calculate an initial blood half-life of ~11.25 minutes by fitting a one-phase exponential decay.

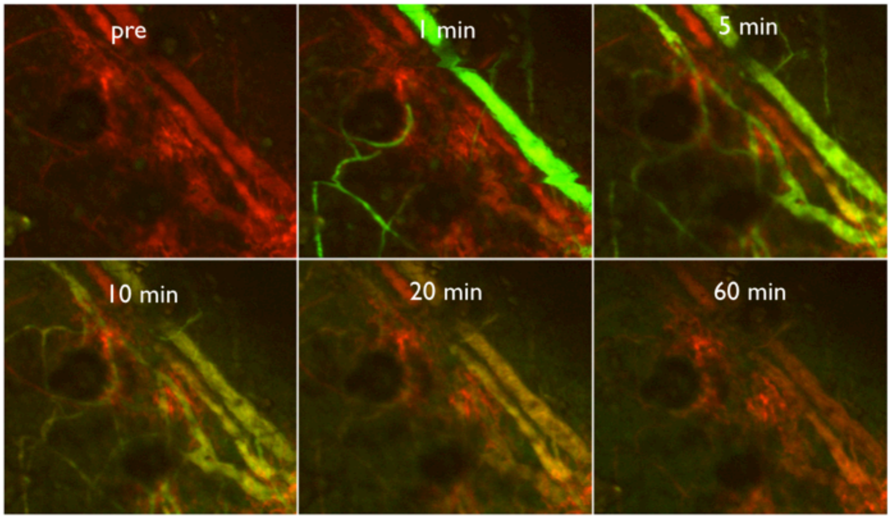

R^2=0.98
Half life= 11.25 min

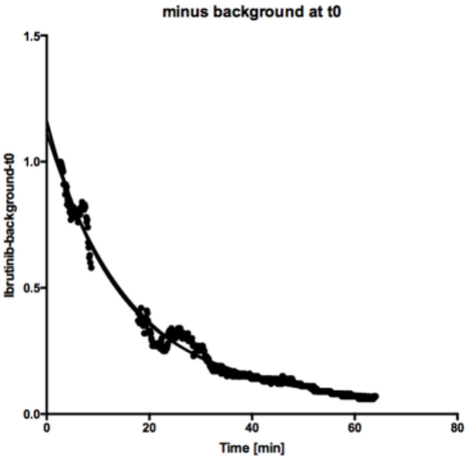

Supplementary Fig. S4. Ex vivo fluorescence imaging with DMSO control or Ibrutinib-BFL (top and bottom of individual organ figures). C57BL/6 mice were IV injected with 150 μL of Ibrutinib-BFL solution [7.5 μL of 10 mM Ibrutinib-BFL solution in DMSO was diluted with 142.5 μL of DMAc:solutol:PBS (v:v:v = 1:1:7.5) mixture (final concentration = 50 μM)] or DMSO control. One hour after injection, animals are euthanized and the organs were collected. Ex vivo fluorescence images of organs were obtained using FMT 2500 system (Perkin Elmer).

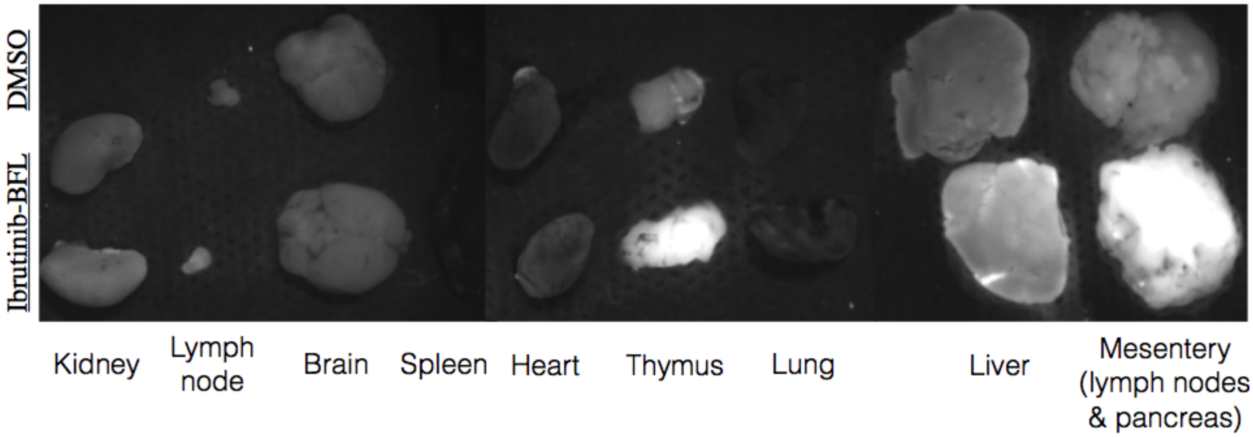

Spectra ^{1}H and ^{13}C of compound 2

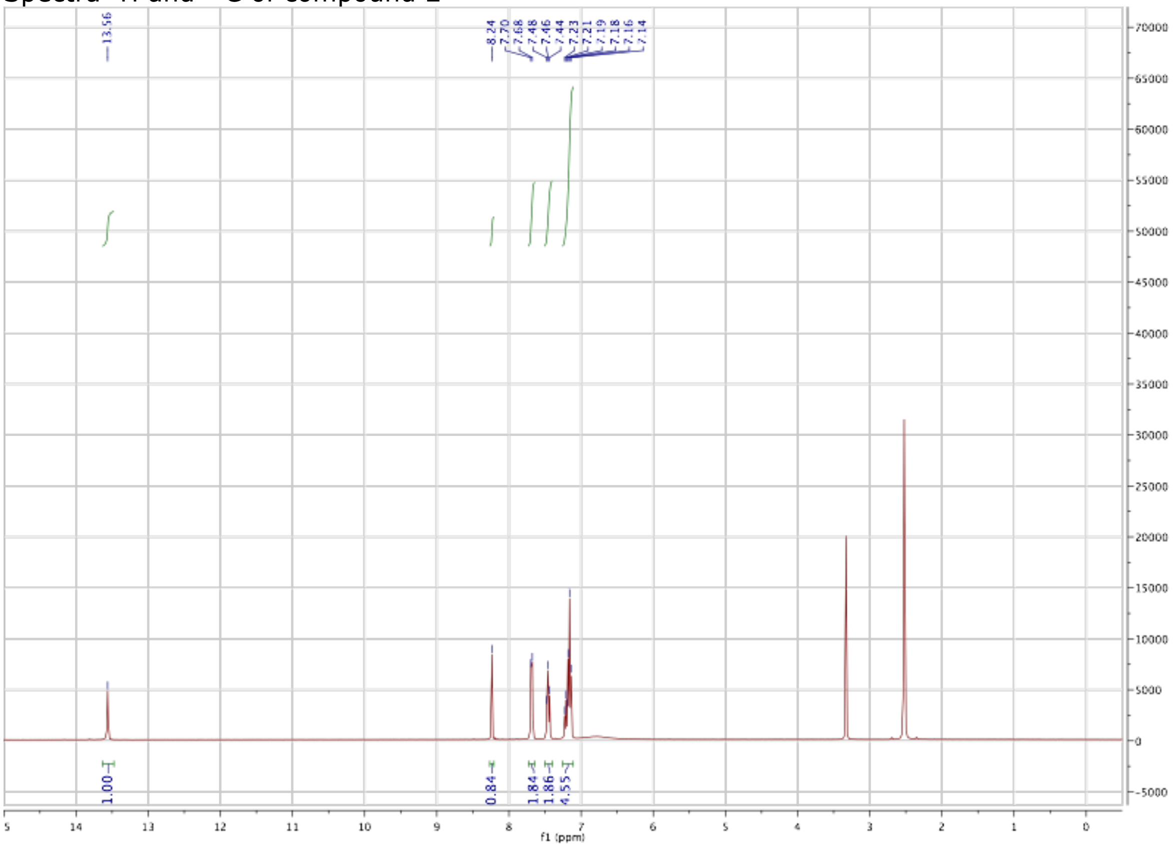

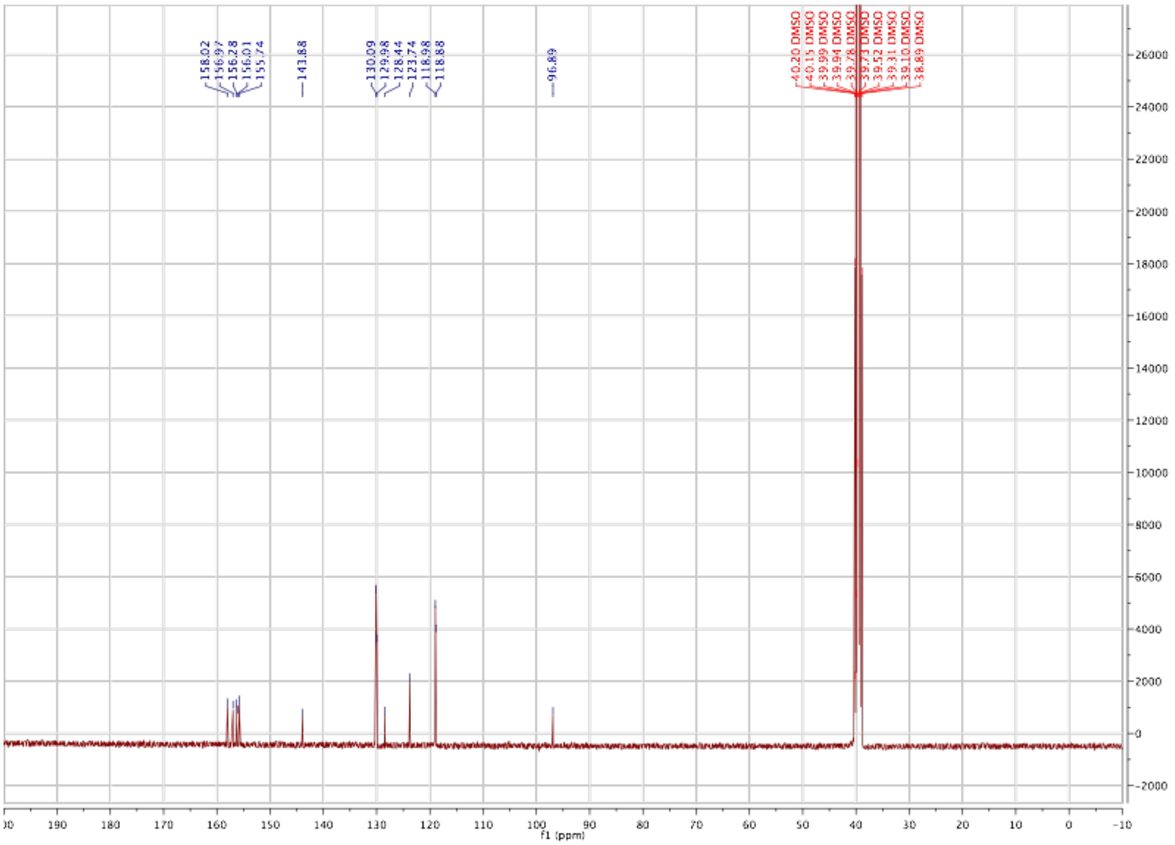

Spectra ^{1}H and ^{13}C of compound 3

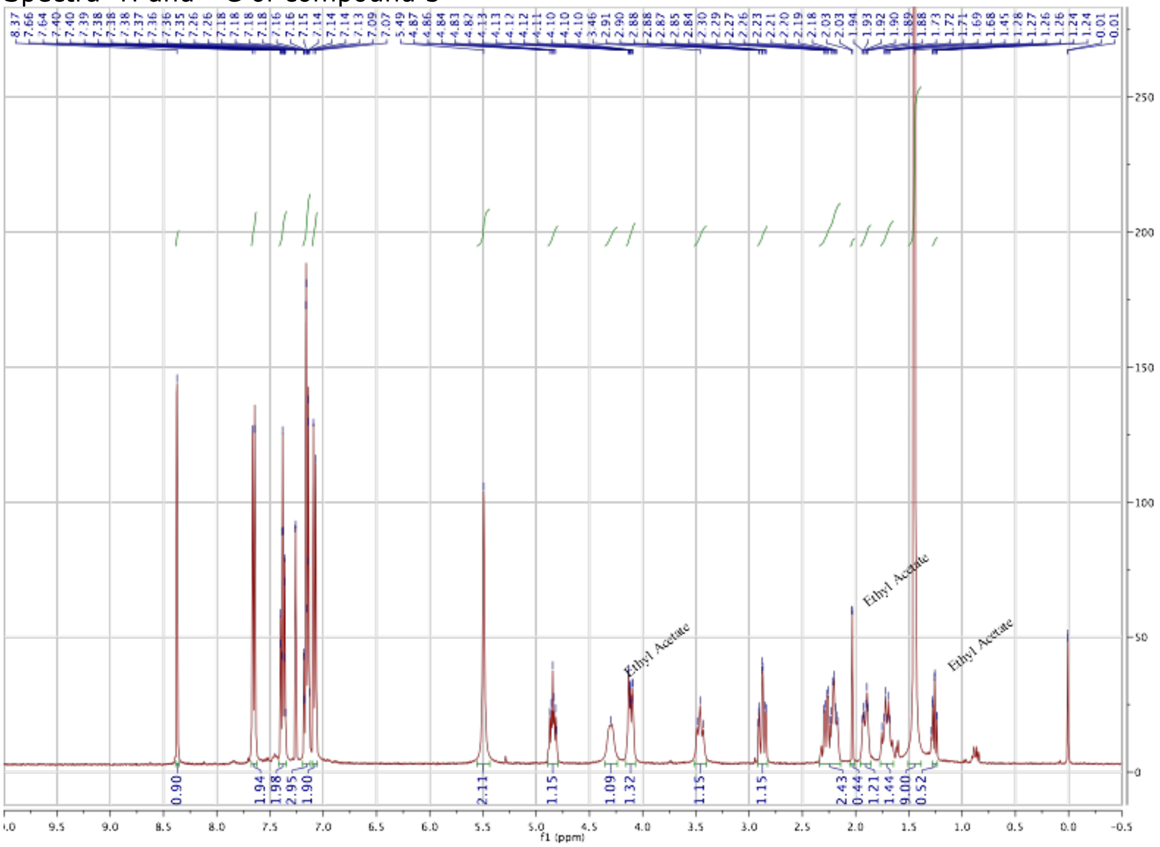

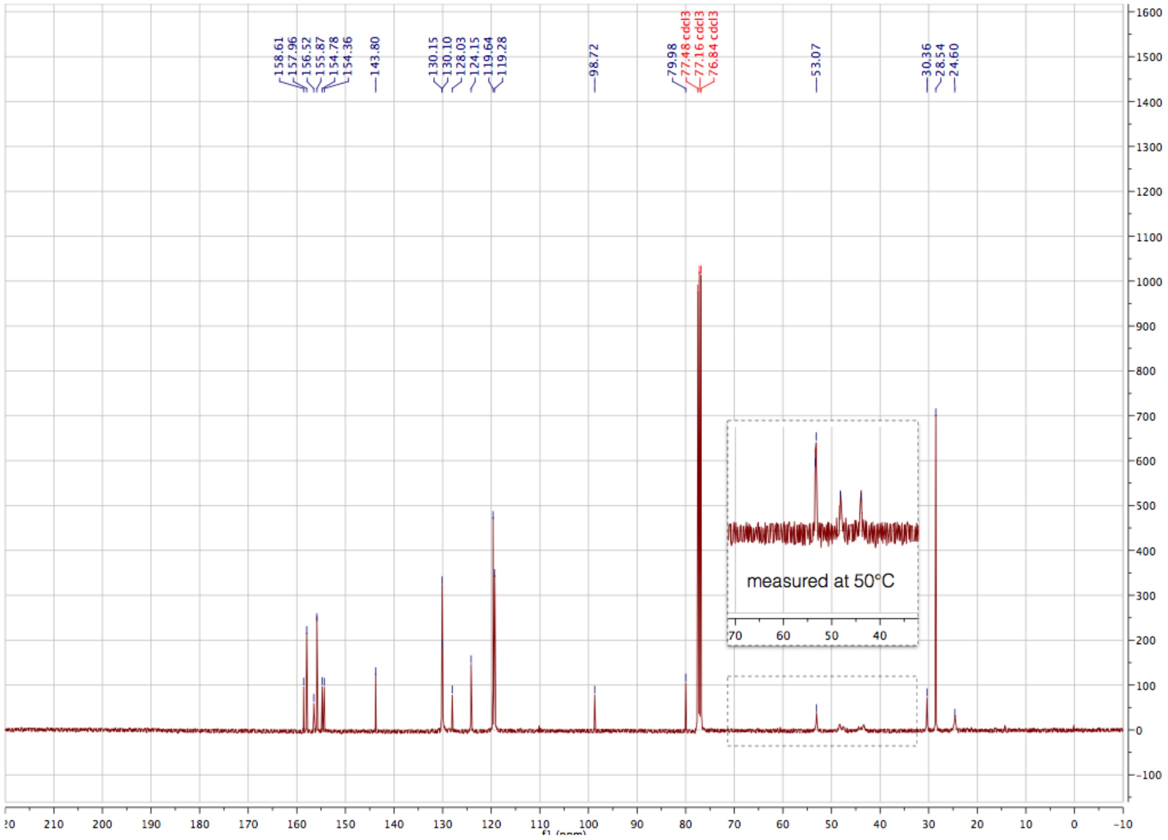

Spectra ^{1}H and ^{13}C of compound 4

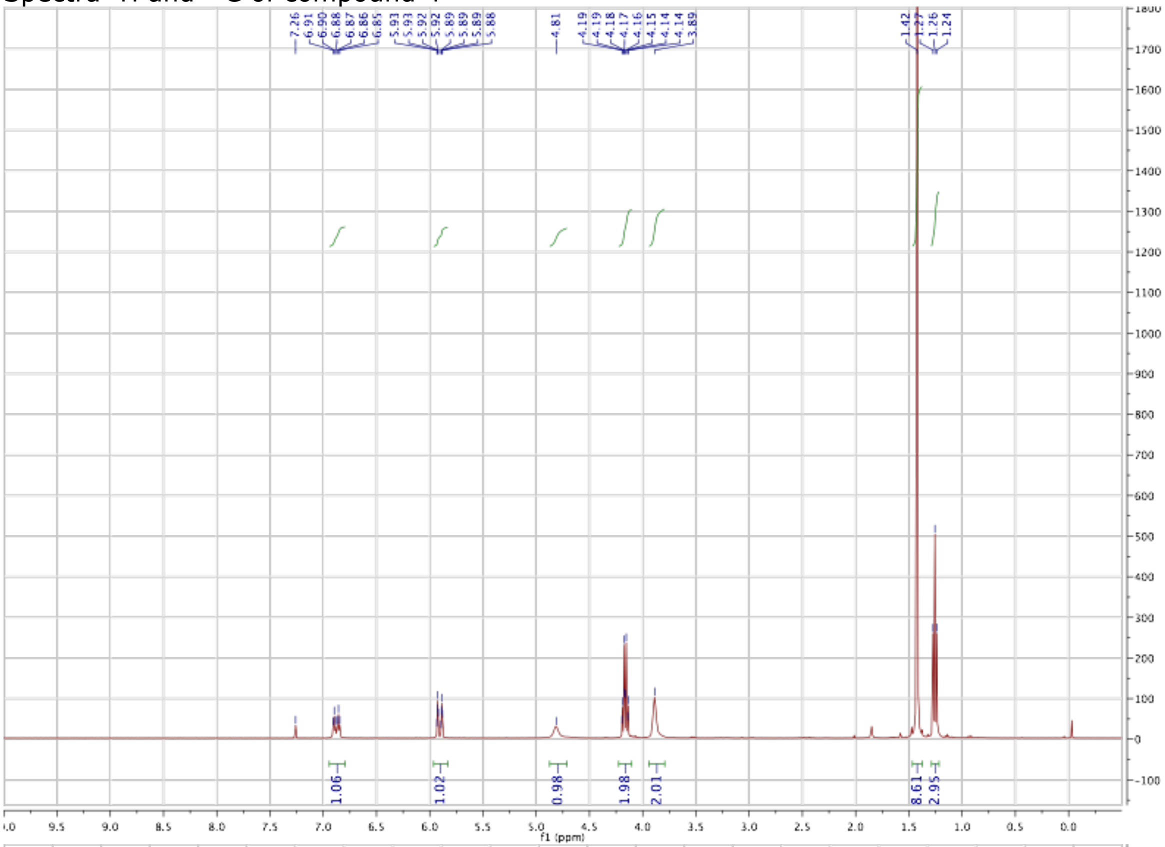

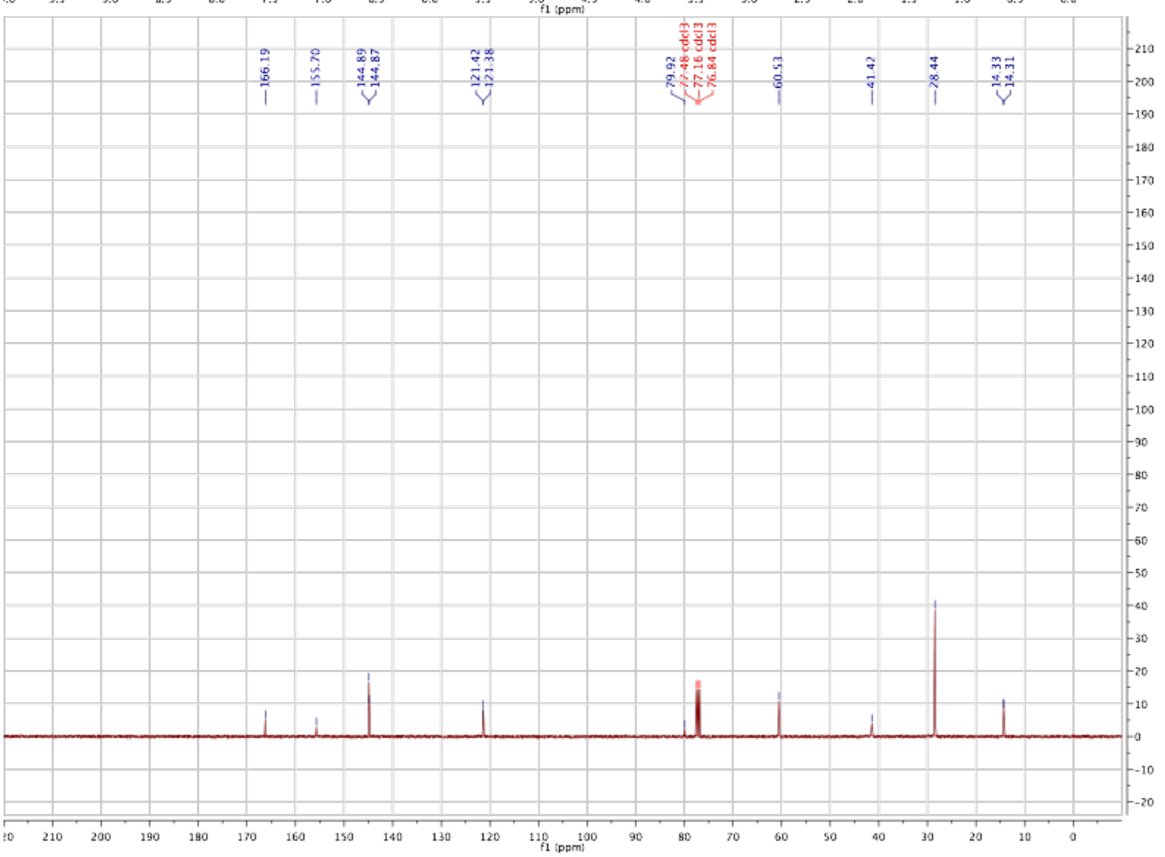

Spectra ^{1}H and ^{13}C of compound 5

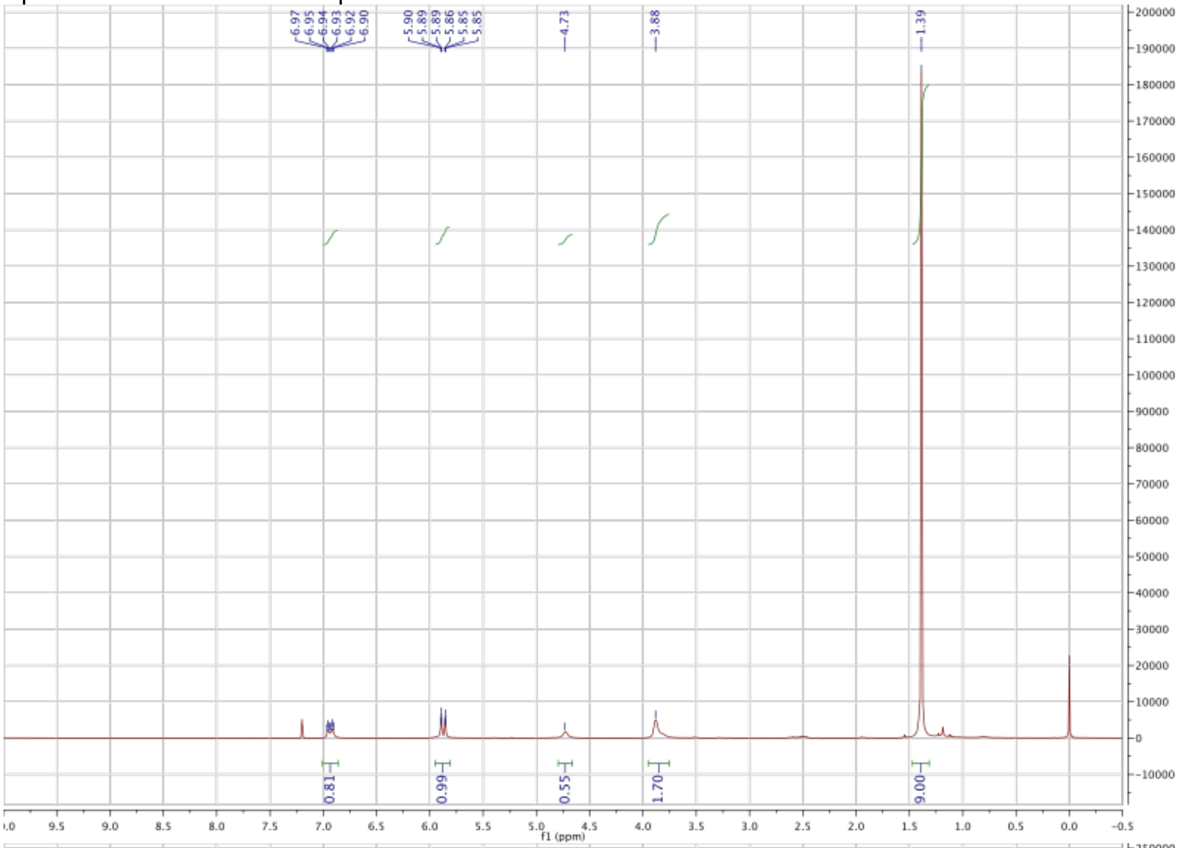

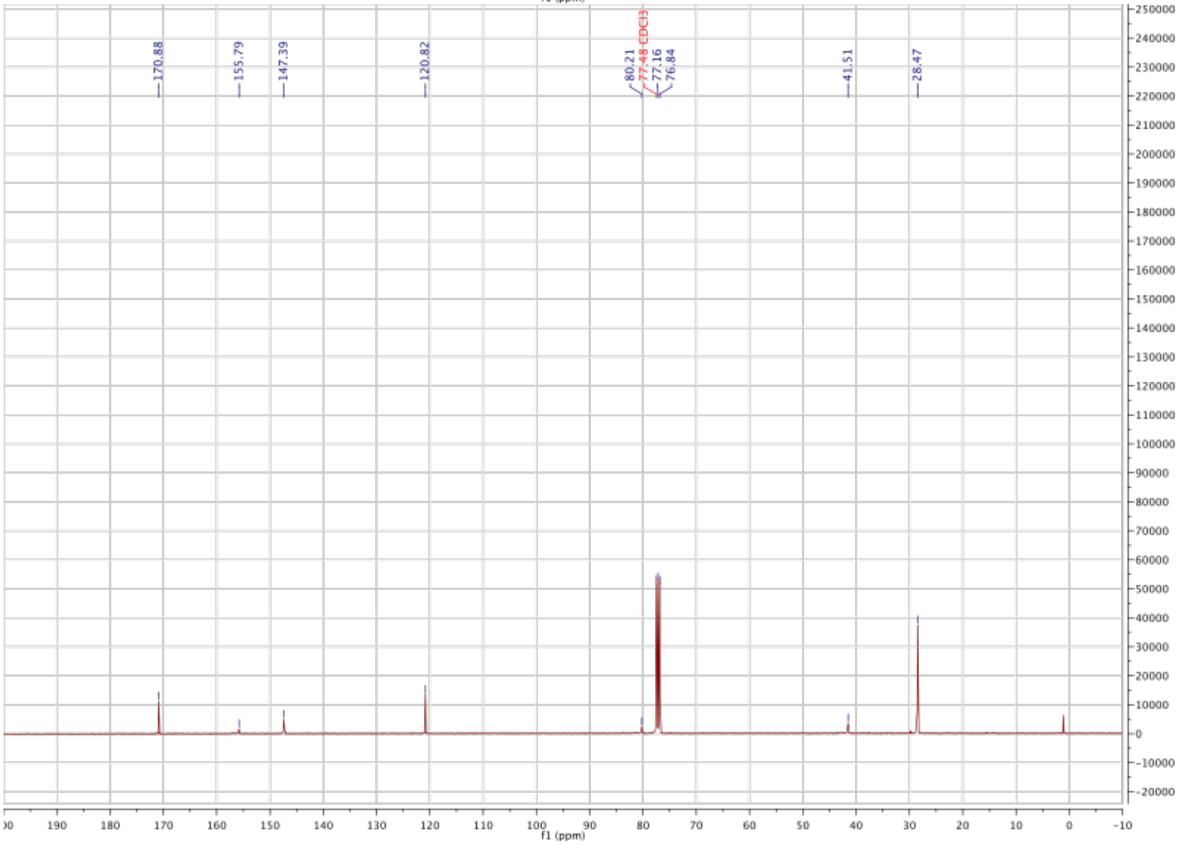

Spectra ^{1}H of compound 6

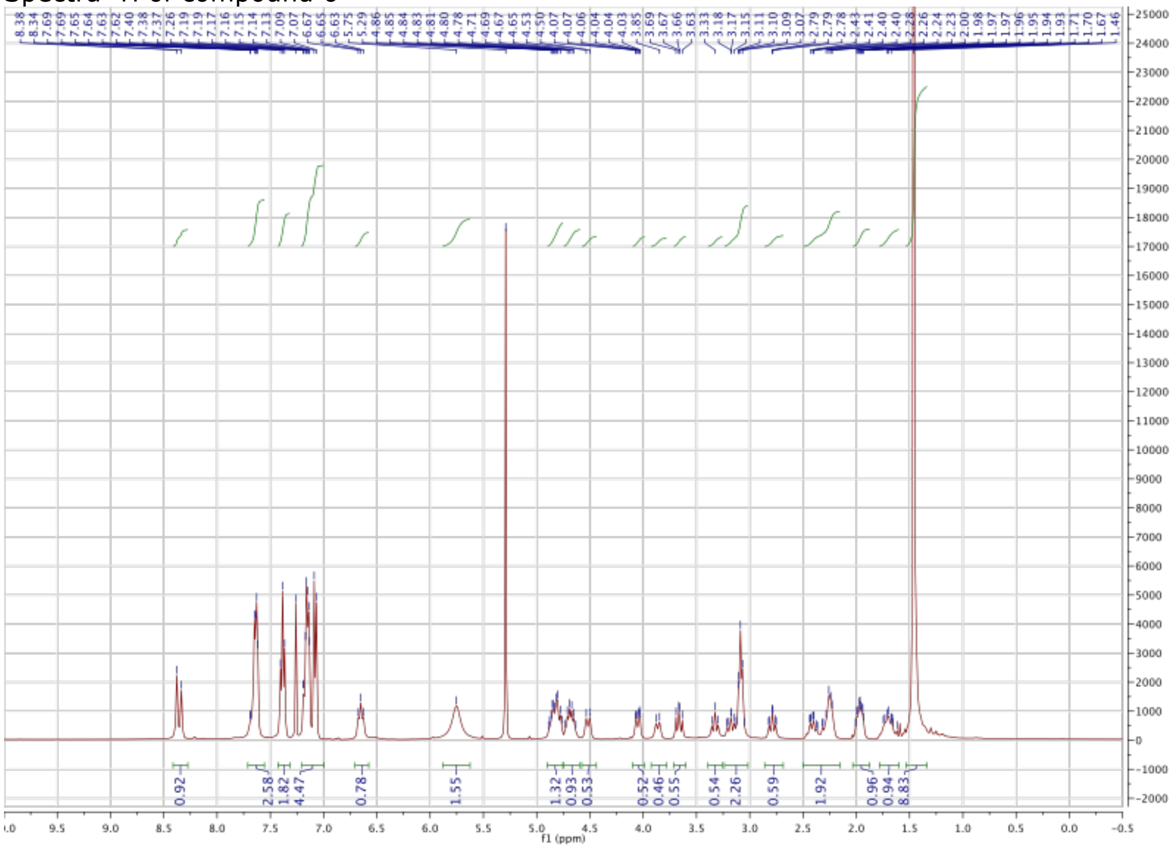

Spectra ^{1}H of compound 7

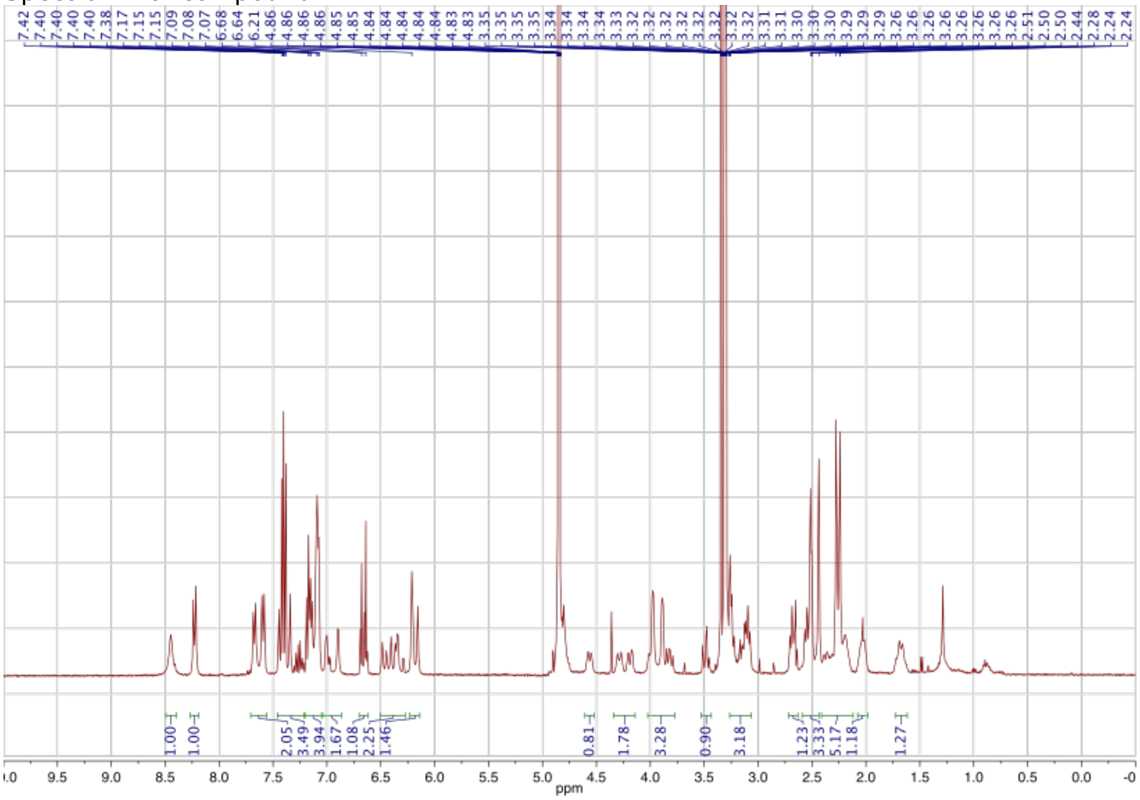

Supplement: Supplementary Information [file srep04782-s1.pdf]
